# Inflammation associated with lung function abnormalities in COVID-19 survivors

**DOI:** 10.1186/s12890-023-02521-5

**Published:** 2023-07-01

**Authors:** Roberto Mancilla-Ceballos, Kathryn M. Milne, Jordan A. Guenette, Arturo Cortes-Telles

**Affiliations:** 1 Internal Medicine Department, Hospital Regional de Alta Especialidad de La Peninsula de Yucatan, Yucatan, Mexico; 2 Department of Medicine, The University of British Columbia, Vancouver, Canada; 3 Centre for Heart Lung Innovation, Providence Research, The University of British Columbia and St. Paul’s Hospital, Vancouver, Canada; 4 Department of Physical Therapy, The University of British Columbia, Vancouver, Canada; 5 Respiratory Diseases Clinic, Hospital Regional de Alta Especialidad de La Peninsula de Yucatan, Yucatan, Mexico

**Keywords:** Pulmonary function, Restrictive abnormalities, COVID-19, Biomarkers, Neutrophil-to-lymphocyte ratio

## Abstract

**Background:**

Activation of inflammatory pathways promotes organ dysfunction in COVID-19. Currently, there are reports describing lung function abnormalities in COVID-19 survivors; however, the biological mechanisms remain unknown. The aim of this study was to analyze the association between serum biomarkers collected during and following hospitalization and pulmonary function in COVID-19 survivors.

**Methods:**

Patients recovering from severe COVID-19 were prospectively evaluated. Serum biomarkers were analyzed from admission to hospital, peak during hospitalization, and at the time of discharge. Pulmonary function was measured approximately 6 weeks after discharge.

**Results:**

100 patients (63% male) were included (age 48 years, SD ± 14) with 85% having at least one comorbidity. Patients with a restrictive spirometry pattern (*n* = 46) had greater inflammatory biomarkers compared to those with normal spirometry (*n* = 54) including peak Neutrophil-to-Lymphocyte ratio (NLR) value [9.3 (10.1) vs. 6.5 (6.6), median (IQR), *p* = 0.027] and NLR at hospital discharge [4.6 (2.9) vs. 3.2 (2.9) *p* = 0.005] and baseline C-reactive protein value [164.0 (147.0) vs. 106.5 (139.0) mg/dL, *p* = 0.083). Patients with an abnormal diffusing capacity (*n* = 35) had increased peak NLR [8.9 (5.9) vs. 5.6 (5.7) mg/L, *p* = 0.029]; baseline NLR [10.0 (19.0) vs. 4.0 (3.0) pg/ml, *p* = 0.002] and peak Troponin-T [10.0 (20.0) vs. 5.0 (5.0) pg/ml, *p* = 0.011] compared to patients with normal diffusing capacity (*n* = 42). Multivariable linear regression analysis identified predictors of restrictive spirometry and low diffusing capacity, but only accounted for a low degree of variance in pulmonary function outcome.

**Conclusion:**

Overexpression of inflammatory biomarkers is associated with subsequent lung function abnormalities in patients recovered from severe COVID-19.

**Supplementary Information:**

The online version contains supplementary material available at 10.1186/s12890-023-02521-5.

## Introduction

Activation of coagulation and inflammatory pathways promotes multiorgan dysfunction in sepsis, acute respiratory distress syndrome (ARDS) and coronavirus disease caused by the SARS-CoV-2 virus (COVID-19) [1]. Survivors of critical illness frequently experience persistent alteration in muscle and cognitive function as well as quality of life [2, 3]. Persistent symptoms, impaired cognitive function, radiographic abnormalities, reduced quality of life, and alterations in pulmonary function including restrictive patterns and reduced diffusing capacity, have been described in patients recovering from COVID-19 [4–6].

Inflammatory markers implicated in the severity and prognosis of COVID-19 include increased Neutrophil-to-Lymphocyte ratio (NLR), C-reactive protein (CRP), and decreased Lymphocyte-to-C-reactive protein ratio (Lym-to-CRP) [7–10]. Particularly, the NLR reflects the immunopathological response and inflammatory state in COVID-19. NLR is associated with increased reactive oxygen species promoting tissue damage, thrombosis, and progression to more severe disease [11, 12] as well as development of ARDS [13]. Persistently increased levels of NLR and CRP have been shown to be related to severity of imaging findings on chest computed tomography in hospitalized patients with COVID-19 [14, 15]. A recent study by Mendez et al. has described an association between female sex, smoking history, and increased D-dimer with reduced diffusing capacity [16]. This work highlights the importance of using biomarkers to identify patients more likely to have impaired lung function. However, the biological mechanisms underpinning these abnormalities and readily available validated biomarkers to identify patients likely to develop impaired lung function remain largely unknown.

The aim of the present study was to examine the relationship between serum biomarkers of inflammation, organ dysfunction, and coagulation throughout hospital stay and at discharge with subsequent pulmonary function test (PFT) findings in patients recovering from COVID-19. We hypothesized that biomarkers of increased inflammation, organ dysfunction, and altered coagulation would be associated with lung function abnormalities at 6-week follow-up in patients recovering from severe COVID-19.

## Methods

### Study design

This was a single-center observational study of patients recovering from severe COVID-19 who performed PFTs 6 weeks after hospital discharge. Severe disease was defined as patients hospitalized with COVID-19 requiring supplemental oxygen (O_2_ > 5 L/min) and prone positioning for at least 12 h per day without invasive mechanical ventilation. This project was approved by the Ethics Committee of the Hospital Regional de Alta Especialidad de la Peninsula de Yucatan (Protocol number 2020–023) and all patients provided written informed consent.

### Patients

All discharged non-critically ill patients who tested positive for SARS-CoV-2 on real-time reverse transcriptase-polymerase chain reaction nasal swabs were eligible for study inclusion. Patients were excluded if laboratory biomarkers included in our study were not collected during hospitalization. Patients were identified through screening of hospital records and invited to participate in the study by telephone 4 to 6 weeks after hospital discharge. All participants subsequently attended the Long-Term Follow-up COVID-19 unit at the Respiratory and Thoracic Surgery Department. All patients provided written informed consent to participate in this study, in compliance with the Helsinki declaration.

### Data collection

Patient data including demographics, laboratory, and pulmonary function test results were collected from medical records. Demographic data included age, sex, body mass index (BMI), and medical history. Laboratory test results were extracted from time of admission to hospital, peak value during hospitalization, and hospital discharge. PFTs including spirometry and diffusing capacity of the lungs for carbon monoxide (D_LCO_), were performed 44 days [interquartile range (IQR) 22] following hospital discharge, according to international guidelines [17, 18] using standard equipment (Ultima PF™ Pulmonary Function System, Medical Graphics, UK or Easy One Pro®, ndd Medical Technologies, Switzerland). A restrictive spirometry abnormality was defined as a forced expiratory volume in 1 s (FEV_1_)/forced vital capacity (FVC) ≥ lower limit of normal (LLN) and FVC < 80% predicted. Abnormal diffusing capacity was defined as D_LCO_ < 80% predicted according to Global Lung Function Initiative reference values [19]. Laboratory tests included complete blood count, CRP, serum ferritin, lactate dehydrogenase (LDH), creatine kinase (CK), creatine kinase-MB (CK-MB), troponin-T, D-dimer, and fibrinogen. NLR and Lym-to-CRP were calculated. The biomarkers were divided into three categories related to: inflammation, organ injury, and coagulation.

### Statistical analysis

Independent t-tests, Mann–Whitney U test, and Fisher’s exact tests were used to compare anthropometric, pulmonary function, and laboratory biomarkers, where appropriate. The association between biomarkers and FVC or D_LCO_ value at follow up was determined using best subset multivariable linear regression. Best subset multivariable linear regression was used as this approach compares all possible models based on available independent variables. Models were used to determine the association between potential inflammatory, organ injury, and coagulation predictor variables and lower FVC (< 80% predicted) and D_LCO_ (< 80% predicted). Peak biomarker values were used in the regression model as these values represented the greatest degree of biomarker abnormality. Independent variables included: NLR peak, CRP peak, Lym-to-CRP nadir, ferritin peak, LDH peak, CK peak, CK-MB peak, troponin-T peak, D-dimer peak, and fibrinogen peak. All models were adjusted for the time between COVID-19 symptom onset and PFT measurement, age, sex, obesity, hypertension, diabetes, and smoking status. A two-tailed *p*-value < 0.05 was considered statistically significant. Results are presented as mean ± standard deviation or median (interquartile range, IQR) for parametric and non-parametric variables, respectively. Statistical analysis was performed using STATA V.13 (Statacorp, College Station, Tx.) and RStudio.

## Results

### Study population

**Fig. 1:**
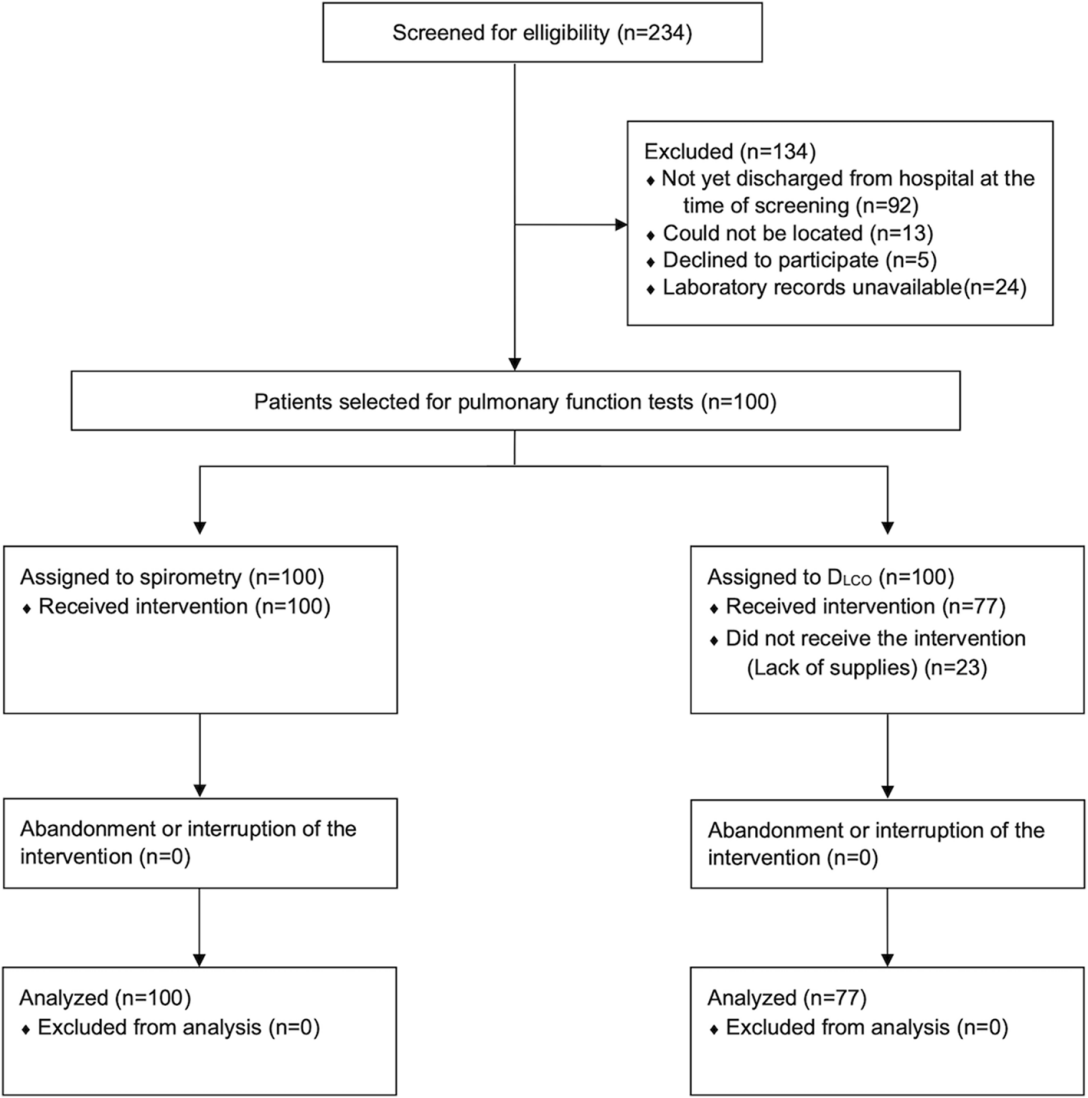
Flow chart showing screening, recruitment, and testing of study participants

Between March 26 and September 30, 2020, a total of 234 patients were identified of which 134 were ineligible (Fig. 1). A total 100 patients hospitalized for severe COVID-19 with complete biomarker data were included in the study and performed pulmonary function tests 6 weeks after discharge. Demographic characteristics of participants are presented in Table 1. The mean age of participants was 48 ± 14 years and 63% were male. Average BMI was consistent with obesity (BMI 33.5 ± 7.2 kg/m^2^) and 85% of participants had at least one comorbidity. The most common comorbidities among participants were obesity (64%), hypertension (26%), and diabetes mellitus (25%). Patients were treated with corticosteroid (equivalent to prednisone 40 mg/day for 10 days) and low molecular weight heparin (equivalent to enoxaparin 1 mg/kg/day while in hospital).

**Table 1:** Participant (*n* = 100) characteristics, peak serum biomarkers during hospitalization, and pulmonary function results at 6 weeks following discharge

Variable	Result
Age (Years)	48 ± 14
Male, n (%)	63 (63%)
Comorbidities, n (%)	85 (85%)
Diabetes	25 (25%)
Hypertension	26 (26%)
Obesity	64 (64%)
Current Smoker	11 (11%)
Pulmonary Function Test	
Spirometry	
FVC, % predicted	79 ± 18
FEV_1_, % predicted	85 ± 19
FEV_1_/FVC, %	87 ± 9
Diffusing Capacity	
D_LCO_, %predicted	88 ± 26*
In-hospital peak value of serum biomarkers	
Inflammatory biomarkers	
Neutrophil-to-Lymphocyte Ratio	8.3 (8.9)
C‐reactive protein (0–5 mg/L)	163 (167)
Minimum Lymphocyte‐to‐C‐reactive protein ratio	34.6 (94.7)
Serum ferritin (18–341 ng/L)	1614 (1124)
Organ-injury biomarkers	
Lactate dehydrogenase (240–480 U/L)	705 (302)
Creatine kinase (39–308 U/L)	110 (156)
Creatine kinase-MB (1–25 U/L)	28 (18)
Troponin-T (0–14 pg/mL)	6 (8)
Coagulation biomarkers	
D-dimer (0–500 ng/mL)	810 (1510)
Fibrinogen (170–254 mg/dL)	789 (267)

Values are presented as mean ± standard deviation for parametric variables, median (interquartile range) for nonparametric variables, or number (%). *n = 77 participants for D_LCO_ measurement

Reference normal values for the laboratory where measurement of biomarker variables was performed are listed in parentheses when available following variable name

Abbreviations: D_LCO_: diffusing capacity of the lungs for carbon monoxide; FEV_1_: forced expiratory volume in 1 s; FVC: forced vital capacity

All participants completed spirometry and 77 participants completed D_LCO_ measurement (Fig. 1). Average results for lung function measures included: FVC: 79 ± 18% predicted, FEV_1_: 85 ± 19% predicted, and FEV_1_/FVC: 87 ± 9%. D_LCO_ was 88 ± 26% predicted, although there was significant variability in results between participants. Survivors of COVID-19 had abnormal peak serum biomarkers during hospitalization related to inflammation, organ injury, and coagulation (Table 1).

### Differences in serum biomarker profile and spirometry

**Fig. 2:**
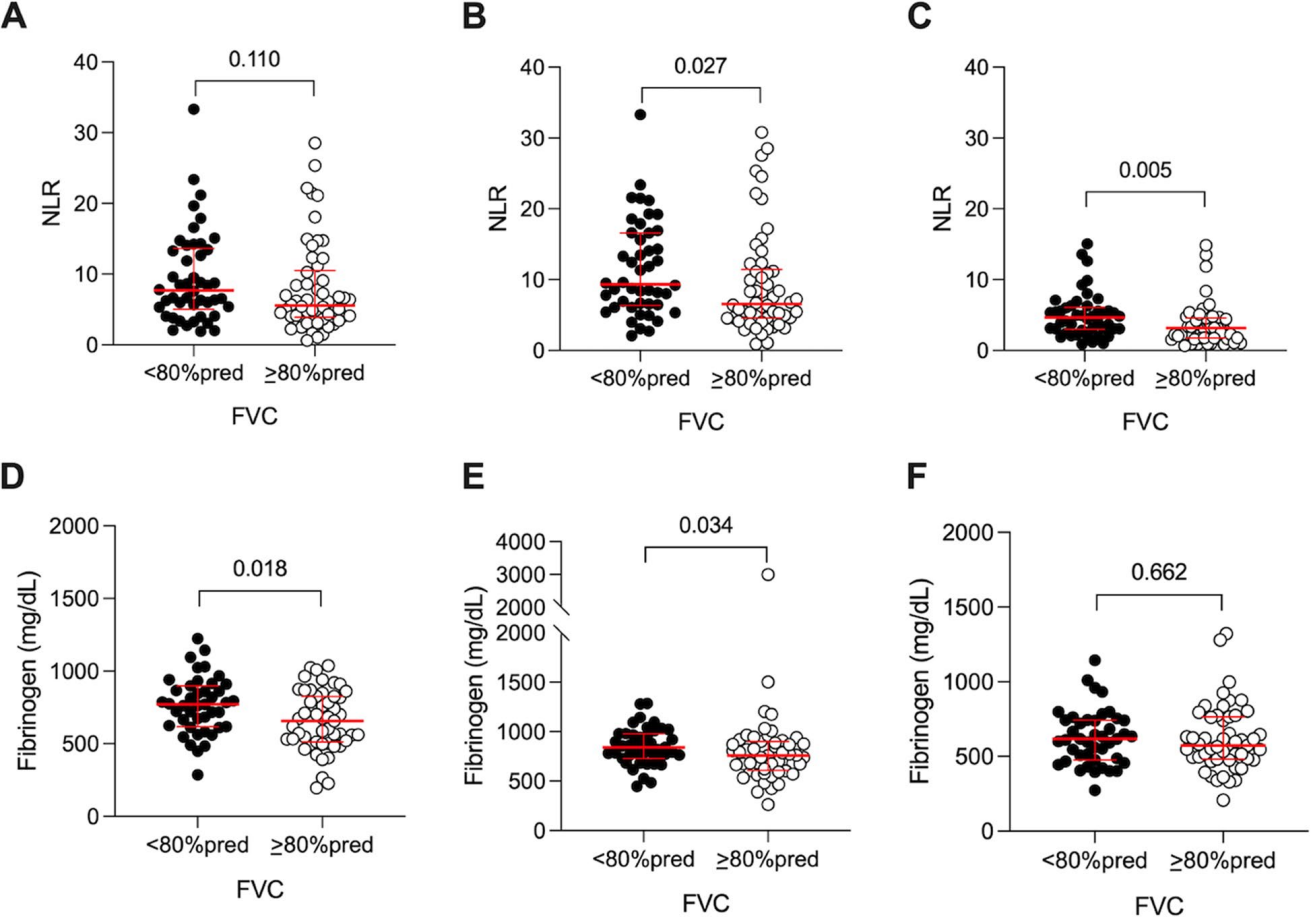
Inflammatory and coagulation biomarker differences during hospitalization between patients with reduced FVC (< 80% predicted) and preserved FVC (≥ 80% predicted) at 6 weeks of recovery from severe COVID-19. A and D: baseline, B and E: peak during hospitalization, C and F: discharge. Abbreviations: FVC: forced vital capacity; NLR: Neutrophil-to-lymphocyte ratio

Differences in serum biomarkers in patients with a restrictive spirometric pattern (FVC < 80% predicted) compared to those with a preserved FVC are summarized in Supplementary Table 1. Patients with a reduced FVC had evidence of increased inflammation in related biomarkers including: peak NLR value [9.3 (10.1) 95% CI 9.5–13.4 vs. 6.5 (6.6), 95% CI 7.4–11.4, *p* = 0.027] and NLR at hospital discharge [4.6 (2.9) 95% CI 4.0–5.9 vs 3.2 (2.9), 95% CI 2.9–4.5, *p* = 0.005], as well as baseline CRP value [164.0 (147.0) 95% CI 136.5–195.9 vs. 106.5 (139.0) 95% CI 106.7–161.1 mg/L, *p* = 0.083). The trajectory of NLR in patients with and without a restrictive spirometric pattern from admission to discharge is shown in Fig. 2 A-C. Increased baseline fibrinogen value [772 (282) 95% CI 705–825 vs. 657 (313) 95% CI 600–716 mg/dl, *p* = 0.018] and peak fibrinogen value [841 (252) 95% CI 793–908 vs. 760 (293) 95% CI 696–911 mg/dl, *p* = 0.034] in patients with reduced FVC were the only significant differences in coagulation related biomarkers (Fig. 2 D-F). There were no significant differences in organ-injury biomarkers in participants with or without a restrictive spirometry pattern.

### Differences in serum biomarkers profile and diffusing capacity

**Fig. 3:**
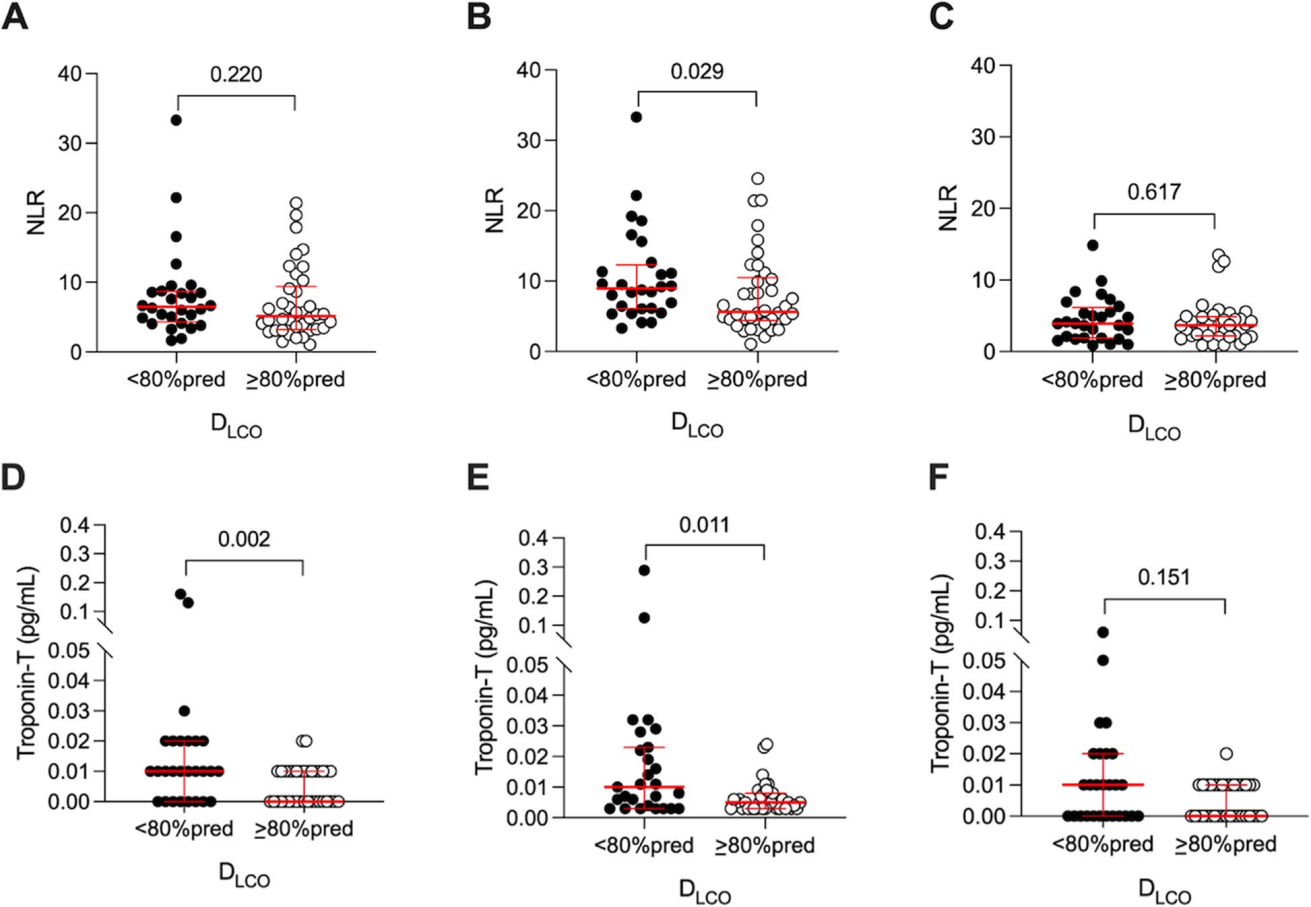
Inflammatory and organ-injury biomarker differences during hospitalization between patients with reduced D_LCO_ (< 80% predicted) and preserved D_LCO_ (≥ 80% predicted) at 6 weeks of recovery from severe COVID-19. A and D: baseline, B and E: peak during hospitalization, C and F: discharge. Abbreviations: D_LCO_: diffusing capacity of the lungs for carbon monoxide; NLR: Neutrophil-to-lymphocyte ratio

Comparison of serum biomarkers in participants with and without decreased diffusing capacity are summarized in Supplementary Table 2. Compared to patients with preserved diffusing capacity (D_LCO_ >80% predicted), peak NLR value [8.9 (5.9) 95% CI 8.0–13.1 vs. 5.6 (5.7), 95% CI 6.2–9.9 *p* = 0.029] and peak CRP value [197.0 (156.0) 95% CI 161.3–238.2 vs. 145.1 (193.0) 95% CI 118.0–189.8 mg/L, *p* = 0.068] were higher in participants with impaired diffusion capacity, suggestive of greater inflammation in this group. In biomarkers related to organ injury, the baseline troponin-T value [10.0 (19.0) 95% CI 10.0–40.0 vs. 4.0 (3.0) 95% CI 0.0–10.0 pg/ml, *p* = 0.002] and the peak troponin-T value [10.0 (20.0) 95% CI 4.0–49.0 vs. 5.0 (5.0) 95% CI 5.0–8.0 pg/ml, *p* = 0.011], were significantly higher in patients with abnormal pulmonary diffusion. The trajectory of NLR and troponin-T values throughout hospitalization is shown in Fig. 3. There were no significant differences between biomarkers associated with coagulation.

### Regression analysis

As presented in Table 2, from selected biomarkers, the combination of NLR peak, CRP peak, and ferritin peak values formed the best combination of biomarkers to describe FVC (% predicted) outcome (F 8,85 = 2.48; *p* = 0.018). However, the model only explained up to 11.3% of the variance of FVC. A second regression model was constructed to examine the relationship between biomarkers and reduced D_LCO_ (% predicted) (Table 2). The overall model was not significant in predicting abnormalities in D_LCO_ (F 7,40 = 1.725; *p* = 0.131), and explained only 9.8% of the variance in D_LCO_ outcome.

**Table 2:** Adjusted results of best subset multivariable linear regression for predictors of FVC and D_LCO_

Predictor	Estimate	SE	T	*p*-value
Predictors of FVC (% predicted)				
Intercept	65.400	11.063	5.911	< 0.001
Neutrophil-to-Lymphocyte Ratio peak	-0.614	0.291	-2.107	0.038
C‐reactive protein peak	-0.020	0.022	-0.941	0.349
Ferritin peak	0.002	0.001	1.602	0.113
Age	-0.030	0.141	-0.216	0.830
Sex	3.846	4.175	0.921	0.360
Days from symptom onset to pulmonary function testing	0.259	0.129	2.011	0.048
Obesity	5.052	4.209	1.200	0.233
Smoker	1.671	5.952	0.281	0.780
Predictors of D_LCO_ (% predicted)				
Intercept	84.930	20.330	4.178	< 0.001
D-dimer peak	-0.001	0.001	-1.223	0.229
Troponin-T peak	-89.850	93.790	-0.958	0.344
Age	-0.440	0.297	-1.481	0.146
Sex	9.463	9.625	0.983	0.331
Days from symptom onset to pulmonary function testing	0.272	0.248	1.094	0.281
Obesity	6.517	8.369	0.779	0.441
Smoker	14.170	11.430	1.239	0.222

Model was adjusted for confounders of age, sex, days from symptom onset to PFT measurement, obesity, hypertension, diabetes, and smoking status

## Discussion

The role of biomarkers of inflammation, organ injury, and abnormal coagulation from laboratory tests commonly performed as a part of the routine clinical care of hospitalized patients with COVID-19 and their relationship to abnormal lung function during follow-up was the focus of our study. Overall, our findings demonstrate that biomarkers of increased inflammation in patients with severe COVID-19 are abnormal in those that subsequently develop restrictive spirometry and reduced diffusion abnormalities.

Currently, the follow-up of COVID-19 survivors is focused on identifying and characterizing sequelae of illness during recovery. Multiple reports have confirmed the persistence of symptoms, compromised quality of life [6, 20, 21], radiographic abnormalities [22, 23], and pulmonary function impairments [24, 25] following hospital discharge frequently observed in COVID-19 survivors [26]. Persistent pulmonary function abnormalities in this group of patients includes reduced diffusing capacity and patterns of restriction and/or obstruction [24]. However, the possible pathophysiological mechanisms that may be linked to these alterations in lung function have not been fully elucidated.

Biomarkers derived from frequently used laboratory tests in hospitalized COVID-19 patients have been a focus for both understanding potential mechanisms underlying subsequent abnormal lung function and identifying patients at risk of adverse pulmonary outcomes. Recent work examining the relationship between peak CRP and D-dimer values in hospitalized COVID-19 patients found that higher D-dimer, in combination with female sex and smoking history, was associated with reduced diffusing capacity at approximately 3-months follow up [16]. Our study expands on these observations by describing the trajectory of multiple biomarkers throughout the course of hospitalization and examining both restrictive spirometry and reduced diffusing capacity. We identified that a restrictive spirometry pattern and reduced D_LCO_ during follow up were both associated with increased inflammatory markers during hospitalization. However, abnormal inflammatory biomarkers explained only a small to moderate proportion of the variance in PFT results, suggesting that additional factors contribute to the development of abnormal lung function during recovering from COVID-19.

In study participants recovering from severe COVID-19 illness, 46% of patients had a reduced FVC (< 80% predicted) and normal FEV_1_/FVC, suggestive of a restrictive spirometric pattern. These patients had elevated levels of inflammatory biomarkers during hospitalization including NLR and CRP. Although persistence of a hyperinflammatory state during convalescence from COVID-19 is an area of ongoing research, the findings of the present study demonstrate abnormal markers of inflammation during hospitalization in patients subsequently found to have restrictive spirometry. This suggests that inflammation plays a role in lung injury and subsequent abnormal lung function in COVID-19 survivors [27].

A significant number of patients in our study (45%) had reduced diffusing capacity (D_LCO_ < 80% prediction), consistent with other reported cohorts [24, 25]. Similar to our findings in those with restrictive spirometry, increased NLR was significantly associated with impaired gas exchange. Patients with low D_LCO_ also had significantly increased troponin-T values. Unlike the results of Mendez et al. we did not observe an association between D-dimer and reduced diffusing capacity [16]. This may be related to the significant variability of D-dimer results between individuals observed in both studies. This underscores that biomarkers both sensitive and specific for predicting impaired lung function will provide the greatest clinical value in prioritizing the follow up of patients with COVID-19.

D_LCO_ measures the transfer of oxygen from the alveolar space to hemoglobin contained in erythrocytes located in the pulmonary capillaries [28]; any process that affects oxygen transfer will result in measurement abnormalities (either interstitial, circulatory, or hematologic disruptions). The relationship between D_LCO_ and alterations in cardiac function is known to involve the alveolar-capillary membrane and capillary blood volume involved in gas exchange [29, 30]. In COVID-19, thrombotic microangiopathy and interstitial abnormalities [5, 31] have been identified, which together with myocardial involvement, likely contribute to observed alterations in D_LCO_. Impaired gas exchange may therefore result from a dual process culminating in the destruction of the lung parenchyma [32] and thickening of the alveolar walls [33]. Importantly, we did not find evidence of abnormal biomarkers for coagulation dysfunction in patients with reduced D_LCO_. These findings suggest that although plausible physiologic abnormalities that could contribute to low D_LCO_ have been identified in COVID-19, further work is required to uncover the precise mechanisms for development of persistent impaired oxygen transfer.

Limitations of our study include that we were unable to measure specific inflammatory cytokines (IL-6) in our study. Due to resource constraints, it was not possible to measure D_LCO_ in all patients. We additionally excluded hospitalized patients that did not have measurement of the biomarkers included in our study, which may introduce potential bias to our results. Radiographic data was not included in our study as imaging protocols were not standardized, limiting our ability to make quantitative imaging comparisons. Our included patient population tended to be older males and therefore may not be generalizable to younger females. We were not able to specifically exclude patients with diagnosed pulmonary embolism or deep vein thrombosis from our study and this could impact our analysis of coagulation abnormalities observed in our results. We describe associations between biomarkers and reduced FVC and D_LCO_, assuming the premise that these patients did not have impaired lung function prior to COVID-19 infection as pre-existing lung function was not available to confirm this assertion. Finally, although we present biomarker data collected at hospital admission, these results may be influenced by lead time bias.

In conclusion, our results demonstrate that biomarkers of increased inflammation, organ dysfunction, and altered coagulation were related to a restrictive spirometric pattern and impaired diffusing capacity; however, biomarkers included in our study explained a small proportion of the variance in FVC or D_LCO_. Uncovering a potential causal relationship between increased inflammation and impaired lung function may provide a useful therapeutic target in the follow-up of patients with severe COVID-19. Recently, an observational study of 30 COVID-19 survivors with persistent inflammatory interstitial lung disease (predominantly organizing pneumonia) and elevated inflammatory biomarkers treated with a short 3-week course of tapering oral corticosteroid, demonstrated improvement of symptoms, recovery of lung function, and radiological improvement of interstitial abnormalities [5]. Further exploring the relationship between markers of inflammation during hospitalization and other factors that lead to the development of impaired lung function in convalescent patients with severe COVID-19 is urgently needed. Additional research is needed to examine risk stratification models that include biomarkers and other factors in better predicting adverse pulmonary function outcomes.

## Supplementary Information

**Additional file1: Supplementary Table 1**. Comparative analysis of abnormalities in participants with and without a restrictive spirometric pattern.

## Data Availability

All data generated or analysed during this study are included in this article and/or its supplementary material files. The datasets used and/or analysed during the current study are available from the corresponding author on reasonable request.

## Abbreviations

SARS-CoV2: Severe acute respiratory syndrome coronavirus 2
COVID-19: Coronavirus disease 2019
ARDS: Acute respiratory distress syndrome
Lym-to-CRP: Lymphocyte-to-C-reactive protein ratio
NLR: Neutrophil to lymphocyte ratio
CRP: C-reactive protein
LDH: Lactate dehydrogenase
CK: Creatine kinase
CK-MB: Creatine kinase-MB
PFT: Pulmonary function test
D_LCO_: Pulmonary diffusing capacity for carbon monoxide
LLN: Lower limit of normality
FEV_1_: Forced expiratory volume in 1 s
FVC: Forced vital capacity
