# Supplementary material for: Inflammation associated with lung function abnormalities in COVID-19 survivors
Source: BMC Pulm Med. 2023 Jul 1;23:235. doi: 10.1186/s12890-023-02521-5 (PMC10314600; doi:10.1186/s12890-023-02521-5)
Supplement: Supplementary file 1 — Additional file1: Supplementary Table 1. Comparative analysis of abnormalities in participants with and without a restrictive spirometric pattern. [file 12890_2023_2521_MOESM1_ESM.docx]

ONLINE SUPPLEMENT

Inflammation associated with lung function abnormalities in COVID-19 survivors

Roberto Mancilla-Ceballos^1*^

Kathryn M. Milne^2,3*^

Jordan A. Guenette^3,4^

Arturo Cortes-Telles^5^

^1^Internal Medicine Department, Hospital Regional de Alta Especialidad de la Peninsula de Yucatan, Yucatan, Mexico.

^2^Department of Medicine, The University of British Columbia, Vancouver, Canada

^3^Centre for Heart Lung Innovation, Providence Research, The University of British Columbia and St. Paul’s Hospital, Vancouver, Canada

^4^Department of Physical Therapy, The University of British Columbia, Vancouver, Canada

^5^Respiratory Diseases Clinic, Hospital Regional de Alta Especialidad de la Peninsula de Yucatan, Yucatan, Mexico.

*Indicates co-first-authorship

**Supplementary Table 1**. Comparative analysis of abnormalities in participants with and without a restrictive spirometric pattern.

| **Variable** | **FVC < 80%p**  **(n=46)** | **FVC ≥ 80%p**  **(n=54)** | **p-value** |
| --- | --- | --- | --- |
| **Inflamatory biomarkers** |  |  |  |
| **Neutrophil-to-Lymphocyte ratio** | | | |
| Baseline | 7.7 (8.1) | 5.5 (6.3) | 0.110 |
| Peak | 9.3 (10.1) | 6.5 (6.6) | 0.027 |
| Discharge | 4.6 (2.9) | 3.2 (2.9) | 0.005 |
| **C-reactive protein** (0-5 mg/L) | | | |
| Baseline | 164.0 (147.0) | 106.5 (139.0) | 0.083 |
| Peak | 170.0 (167.0) | 145.1 (168.6) | 0.153 |
| Discharge | 44.5 (63.5) | 34.0 (49.0) | 0.346 |
| **Lymphocyte-to- C‐reactive protein ratio** | | | |
| Baseline | 6.4 (10.0) | 13.2 (19.5) | 0.106 |
| Minimum value | 5.2 (6.9) | 8.9 (14.8) | 0.259 |
| Discharge | 33.3 (55.7) | 49.9 (97.0) | 0.341 |
| **Serum Ferritin** (18-341 ng/L) | | | |
| Baseline | 1315 (1146) | 1421 (1178) | 0.742 |
| Peak | 1665 (1091) | 1575 (1177) | 0.842 |
| Discharge | 960 (1135) | 1017 (720) | 0.799 |
| **Organ-Injury Biomarkers** |  |  |  |
| **Lactate dehydrogenase** (240-480 U/L) | | | |
| Baseline | 657 (292) | 653 (339) | 0.602 |
| Peak | 749 (231) | 686 (359) | 0.361 |
| Discharge | 463 (258) | 476 (215) | 0.805 |
| **Creatine kinase** (39-308 U/L) | | | |
| Baseline | 80 (99) | 105 (107) | 0.381 |
| Peak | 105 (154) | 121 (156) | 0.480 |
| Discharge | 44 (47) | 58 (67) | 0.081 |
| **Creatine kinase-MB** (1-25 U/L) | | | |
| Baseline | 21 (13) | 22 (12) | 0.754 |
| Peak | 31 (23) | 26 (16) | 0.149 |
| Discharge | 19 (10) | 18 (8) | 0.488 |
| **Troponin T** (0-14 pg/mL) | | | |
| Baseline | 6 (8) | 4 (5) | 0.401 |
| Peak | 7 (12) | 5 (6) | 0.333 |
| Discharge | 6 (6) | 4 (4) | 0.309 |
| **Coagulation factors** |  |  |  |
| **D-dimer** (0-500 ng/mL) | | | |
| Baseline | 605 (1393) | 500 (500) | 0.194 |
| Peak | 893 (1960) | 740 (1055) | 0.592 |
| Discharge | 405 (698) | 360 (680) | 0.414 |
| **Fibrinogen** (170-254 mg/dL) | | | |
| Baseline | 772 (282) | 657 (313) | 0.018 |
| Peak | 841 (252) | 760 (293) | 0.034 |
| Discharge | 617 (267) | 574 (282) | 0.662 |

Values are presented as median (interquartile range). p-values were obtained from the Mann-Whitney U test.

Reference normal values for the laboratory where measurement of biomarker variables was performed are listed in parentheses when available following variable name.

Abbreviations: FVC: forced vital capacity.

**Supplementary Table 2.** Comparative analysis of biomarker profile in participants with and without reduced diffusing capacity.

| **Variable (normal range)** | **D_LCO_ < 80% predicted**  **(n=35)** | **D_LCO_ ≥ 80% predicted**  **(n=42)** | **p-value** |
| --- | --- | --- | --- |
| **Inflammatory biomarkers** | | | |
| **Neutrophil-to-Lymphocyte ratio** | | | |
| Baseline | 6.5 (4.2) | 5.1 (5.8) | 0.220 |
| Peak | 8.9 (5.9) | 5.6 (5.7) | 0.029 |
| Discharge | 3.9 (4.0) | 3.7 (2.6) | 0.617 |
| **C-reactive protein** (0-5 mg/L) | | | |
| Baseline | 177.0 (150.0) | 102.5 (157.9) | 0.079 |
| Peak | 197.0 (156.0) | 145.1 (193.0) | 0.068 |
| Discharge | 48.0 (83.0) | 27.0 (58.3) | 0.089 |
| **Lymphocyte-to-C‐reactive protein ratio** | | | |
| Baseline | 6.5 (9.4) | 13.2 (22.3) | 0.098 |
| Minimum value | 5.2 (5.8) | 9.5 (17.3) | 0.093 |
| Discharge | 26.9 (24.0) | 35.3 (98.7) | 0.344 |
| **Serum ferritin** (18-341 ng/L) | | | |
| Baseline | 1190 (1310) | 1150 (1309) | 0.574 |
| Peak | 1833 (1081) | 1585 (1298) | 0.597 |
| Discharge | 1094 (960) | 960 (1019) | 0.372 |
| **Organ-injury biomarkers** | | | |
| **Lactate dehydrogenase** (240-480 U/L) | | | |
| Baseline | 695 (352) | 663 (331) | 0.488 |
| Peak | 741 (316) | 766 (346) | 0.780 |
| Discharge | 487 (258) | 505 (306) | 0.909 |
| **Creatine kinase** (39-308 U/L) | | | |
| Baseline | 91 (106) | 104 (110) | 0.926 |
| Peak | 119 (364) | 111 (154) | 0.491 |
| Discharge | 52 (121) | 50 (65) | 0.793 |
| **Creatine kinase-MB** (1-25 U/L) | | | |
| Baseline | 23 (19) | 22 (11) | 0.478 |
| Peak | 27 (21) | 24 (11) | 0.327 |
| Discharge | 17 (16) | 19 (7) | 0.669 |
| **Troponin-T** (0-14 pg/mL) | | | |
| Baseline | 10.0 (19.0) | 4.0 (3.0) | 0.002 |
| Peak | 10.0 (20.0) | 5.0 (5.0) | 0.011 |
| Discharge | 6.0 (16.0) | 4.0 (3.0) | 0.151 |
| **Coagulation biomarkers** | | | |
| **D-dimer** (0-500 ng/mL) | | | |
| Baseline | 668 (1026) | 488 (421) | 0.064 |
| Peak | 1281 (1946) | 625 (1285) | 0.106 |
| Discharge | 516 (684) | 345 (581) | 0.084 |
| **Fibrinogen** (170-254 mg/dL) | | | |
| Baseline | 762 (338) | 675 (293) | 0.494 |
| Peak | 868 (293) | 787 (235) | 0.278 |
| Discharge | 600 (239) | 624 (230) | 0.518 |

Values are presented as median (interquartile range). p-values were obtained from the Mann-Whitney U test.

Reference normal values for the laboratory where measurement of biomarker variables was performed are listed in parentheses when available following variable name.

Abbreviations: D_LCO_: diffusing capacity of the lungs for carbon monoxide.
